# Use of Auricular Acupressure to Improve the Quality of Life in Diabetic Patients with Chronic Kidney Diseases: A Prospective Randomized Controlled Trial

**DOI:** 10.1155/2014/343608

**Published:** 2014-12-10

**Authors:** Shaoqing Wang, Zhaohui Chen, Ping Fu, Li Zang, Li Wang, Xi Zhai, Fang Gao, Aijing Huang, Yao Zhang

**Affiliations:** ^1^Nephrology Department, West China Hospital of Sichuan University, No. 37 Guo Xue Xiang, Chengdu, Sichuan 610041, China; ^2^Department of Integration of Traditional Chinese and Western Medicine, The First Affiliated Hospital of Chengdu Medical College, Chengdu, Sichuan 610513, China; ^3^Nephrology Department, The First Affiliated Hospital of Chengdu Medical College, Chengdu, Sichuan 610513, China

## Abstract

*Background*. Diabetic patients with chronic kidney disease (CKD) suffer from low quality of life (QOL). We aim to assess the effectiveness of auricular acupressure for QOL improvement in these patients. *Materials and Methods*. Sixty-two participants were randomly assigned to an auricular or a control arm in a randomized controlled trial. Participants in the auricular arm were instructed to perform auricular acupressure 3–5 times per day for 3 months, when they were receiving conventional treatments. Participants in the control arm received conventional treatments only. The primary outcome was the summarized score of Kidney Disease and Quality of Life Short-Form (KDQOL-SF) at 3 months after randomization. The secondary outcomes included the 36-Item Short Form Health Survey (SF-36), glycosylated hemoglobin (HbA1c), and estimated glomerular filtration rate (eGFR). *Results*. The summarized KDQOL differed significantly between the acupressure (76.6, 95% CI, 72.2 to 81.0) and the control group (61.8, 95% CI, 57.7 to 65.9). Similar results were found in the SF-36 scores. HbA1c and eGFR were not found to be significantly different between the arms and neither were the adverse events. *Conclusion*. Auricular acupressure was well tolerated in diabetic patients with chronic kidney diseases receiving hemodialysis. Future research is needed to confirm these results.

## 1. Introduction

There will be 300 million diabetic patients in the year 2025. The countries with the largest number of diabetic patients will be India, China, and the United States [1]. Diabetes has become a major health problem in China; the prevalence of diabetes and prediabetes was 9.7%–11.6% and 15.5%–50.1%, respectively, accounting for 92.4 million adults with diabetes and 148.2 million adults with prediabetes [2, 3]. Twenty-five percent of the patients with type 2 diabetes developed microalbuminuria after being diagnosed for 10 years [4]. This high prevalence of albuminuria is alarming and indicates an impending pandemic of diabetic kidney diseases in Asia, with its potential economic consequences [5, 6].

Diabetic patients with chronic kidney diseases often report lower quality of life (QOL) than healthy people, and the diabetes-related complications were the major causes [7]. However, few treatment solutions are available to improve QOL in these patients. Auricular acupressure is a noninvasive and self-manageable method of stimulating the auricular points through pressing by hands and fingers, which has been used for hundreds of years in China [8]. As stated by acupressure theories, the whole human body is reflected in the ears, so various symptoms or conditions could be managed through stimulating a set of auricular points [9]. Auricular acupressure was effective for patients with overweight and high serum lipid problems [10], and thus it was hypothesized that auricular acupressure might be effective for treatment of insulin resistance [11], which was a high-risk predictor for type 2 diabetes [12]. Auricular acupressure was also effective for improving antioxidative system in high-risk diabetes, which would help to prevent diabetic complications [13]. Additionally, auricular acupressure could be helpful to symptom management of end-stage renal diseases, including relieving fatigue and improving activity of daily living [14, 15], which were important for maintaining QOL in these patients. Recent studies indicated that auricular acupressure was effective for improvement of QOL in patients with ischemic heart diseases, breast cancer, and constipation [16–18]. But few studies were conducted to answer the question whether auricular acupressure is also effective for improving QOL in diabetic patients with chronic kidney diseases, when they are receiving hemodialysis. Therefore, we conducted a prospective randomized controlled trial to answer this question.

## 2. Methods 

### 2.1. Study Overview

**Figure 1:**
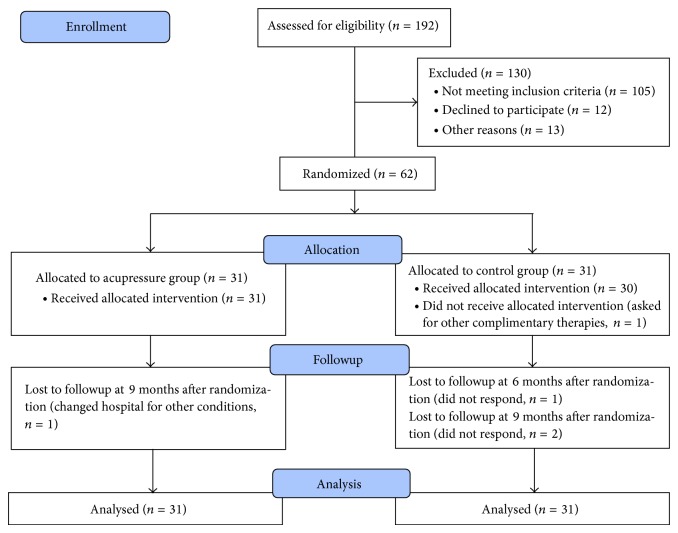
Flow chart of this study.

This study is a prospective randomized controlled trial with an intervention and a control arm. The study was designed and conducted according to the SPIRIT statement (http://www.spirit-statement.org/) [19]. From August, 2012, to March, 2013, a total of 62 diabetic patients with chronic kidney diseases were included in the nephrology department in the first affiliated hospital of Chengdu medical college, to study whether auricular acupressure is effective for improving quality of life (QOL) in these patients. The patients were randomly allocated into 2 arms, the auricular acupressure arm (the acupressure arm) and the conventional treatment control arm (the control arm). In the acupressure arm, the participants were educated to press the auricular points 3 to 5 times in each day for 3 months, and each point was pressed for 2 minutes each time. During these 3 months, the participants also received conventional treatments such as hemodialysis and antidiabetic treatments. In the control arm, participants received only conventional treatments. The participants were followed up for 6 months after they managed the 3-month auricular acupressure. The primary aim of this study is to evaluate the effectiveness of auricular acupressure for improving disease specific QOL, which was assessed using Kidney Disease and Quality of Life Short-Form (KDQOL-SF). We also assessed the overall QOL using the 36-Item Short Form Health Survey (SF-36). Both KDQOL-SF and SF-36 were assessed at baseline and 3, 6, and 9 months after randomization. The control of blood sugar was measured through glycosylated hemoglobin (HbA1c), while the kidney function was measured through estimated glomerular filtration rate (eGFR). HbA1c and eGFR were examined at baseline and 3 months after randomization. The protocol of this study was approved by institutional review boards of Chengdu medical college, and it was registered at the website http://cyfyy.cmc.edu.cn/, with a registered number of CMC20110108.  Figure 1 shows the flow chart of the study, which conforms to the CONSORT statement.

### 2.2. Participants

Patients were included if they were (1) diagnosed as type 2 diabetes [20, 21]; (2) >50 years of age; (3) classified as chronic kidney disease stages 3–5 (GFR <59 mL/min/1.73 m^2^); (4) receiving hemodialysis treatment <1 year. Patients were excluded if they changed the nutritional prescription within 24 hours of treatment or developed intradialytic complications such as severe hypotension requiring fluid resuscitation, hemorrhage, or arrhythmias or had a residual renal clearance of 14.0 mL/min. All patients provided written informed consent to participate in this study prior to randomization.

### 2.3. Randomization and Blinding

Randomization was managed by a statistician who did not know the study aim of arm assignment. He generated the randomization sequences and sealed them in opaque envelopes. When a participant met the inclusion criteria, the research nurse opened an envelope and assigned the participant to the acupressure or the control arm. The participants were not blinded from the assignment to the study arms, because participants in the control arm did not receive any acupressure therapy, and it was not possible to manage blinding. However, the outcome assessors and the statistician were blinded.

### 2.4. Intervention

#### 2.4.1. Conventional Treatments

All participants received conventional treatments, which included hemodialysis treatment and antidiabetic treatments. We used the sustained low-efficiency dialysis (SLED) to give hemodialysis to the participants. SLED was a minor modification to intermittent hemodialysis. In the SLED, the blood was pumped at a rate of 200 mL/min with a dialysate rate of 300 mL for 6 to 12 hours. The SLED was performed with the use of a traditional dialysis machine (Fresenius 4008S and TORAY321), with a frequency of 3 times a week. The SLED and antidiabetic treatments conformed to Clinical Practice Guidelines and Clinical Practice Recommendations for Diabetes and Chronic Kidney Disease [22], released by The National Kidney Foundation Kidney Disease Outcomes Quality Initiative (NKF KDOQI).

#### 2.4.2. Auricular Acupressure

**Figure 2:**
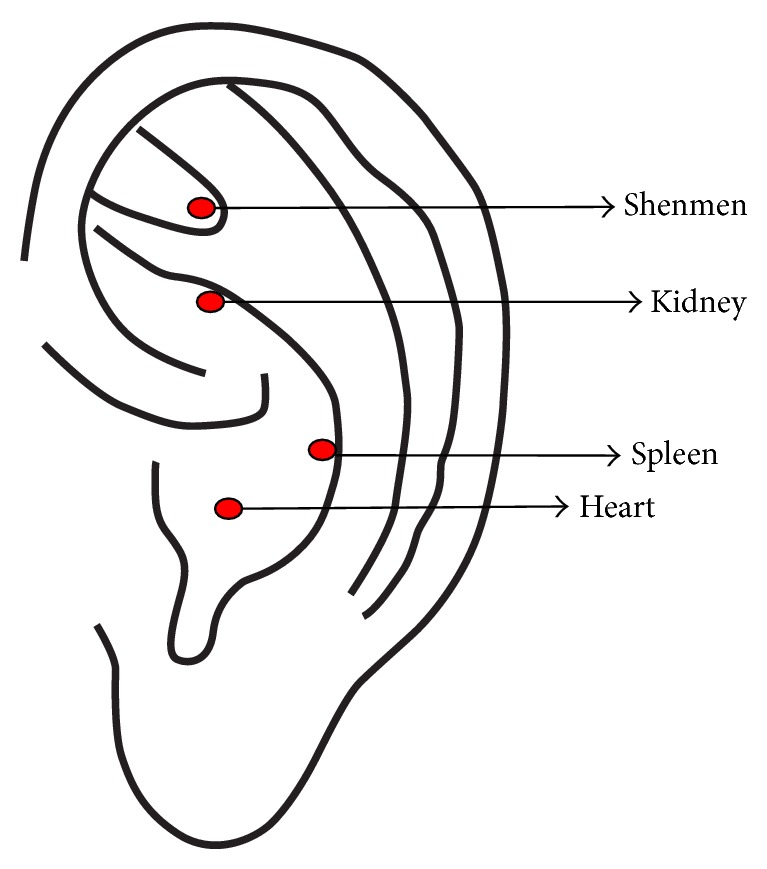
Auricular points selected in this study. The point Shenmen is located at the bifurcating point between superior and inferior antihelix crus, which was used for inducing relaxation, which according to the auricular acupuncture theories is the gate of the soul. The point Kidney was on the lower border of the inferior antihelix crus, which was selected for managing renal diseases. The point Spleen was at the lateral and superior aspect of cavum conchae, which is a nourish point for various conditions; we selected it to manage deficiency due to chronic kidney diseases. The point Heart was in the central depression of cavum conchae, which was selected to induce a feeling of happiness.

The participants in the acupressure arm received self-managed acupressure at 4 auricular points. The 1st point is named Shenmen, which is located at the bifurcating point between superior and inferior antihelix crus. The 1st point was used for inducing relaxation, which according to the auricular acupuncture theories is the gate of the soul [23]. The 2nd point is named Kidney, which is on the lower border of the inferior antihelix crus. This point was selected for managing renal diseases [23]. The 3rd point is named Spleen, which is at the lateral and superior aspect of cavum conchae. The Spleen is deemed to be a nourish point for various conditions; we selected it to manage deficiency due to chronic kidney diseases [23]. The 4th point is named Heart, which is in the central depression of cavum conchae. According to traditional Chinese medicine theories, the heart is the leader of a human's soul and the commander of happiness, so we selected this point [23]. The locations of these 4 points were shown in Figure 2. A practitioner with 10 years of acupuncture experience educated the participants to locate the 4 auricular points and to attach cowherb seeds to these points correctly. The participants pressed cowherb seeds against the 4 auricular points 3 to 5 times a day for 3 months. Each point was pressed for 2 minutes each time. And the participants came to meet the practitioner every 2 weeks to check if they are located at the correct points. And the participants replaced the auricular taps every 3 to 5 days, judged by whether there was pain in the ear. The protocol of auricular acupressure was developed by the acupuncture practitioner through literature review and consultation of acupuncture practitioners.

### 2.5. Outcome Measurements

The primary outcome was the summarized score of Kidney Disease and Quality of Life Short-Form (KDQOL-SF) at the end of auricular acupressure treatment (3 months after randomization). The KDQOL-SF (Rand Health, http://www.rand.org/) was developed to assess the quality of life of individuals with kidney disease and those on dialysis. We used a Chinese version of KDQOL-SF 1.3, which was validated before this study [24]. This KDQOL-SF consisted of 2 parts: the kidney-disease-specific items and the 36-item short form health survey (SF-36) for measuring physical and mental health. We summarized a total score of the kidney-disease-specific items and SF-36 separately according to the methods reported by Hays et al. [25]; the independently total score of the kidney-disease-specific items was considered as the summarized KDQOL-SF and thereafter the primary outcome. The total score of SF-36 was treated as a secondary outcome. The kidney disease-targeted items included 11 areas: symptom/problems (12 items), effect of kidney disease on daily life (8 items), burden of kidney disease (4 items), work status (2 items), cognitive function (3 items), quality of social interaction (3 items), sexual function (2 items), sleep (4 items), social support (2 items), dialysis staff encouragement (2 items), and patient satisfaction (1 item). With the help of research staff, the participants filled in the KDQOL-SF items at baseline and at 3, 6, and 9 months after randomization.

The SF-36 included 8 domains of assessment for participants' quality of life: physical function, physical role limitations, body pain, general health, energy/fatigue, social function, emotional role limitations, and emotional wellbeing. The participants also finished SF-36 at baseline and at 3, 6, and 9 months after randomization. A total score of SF-36 was then calculated, according to the instructions [26].

At baseline and 3 months after randomization, participants took the examination of glycosylated hemoglobin (HbA1c) and estimated glomerular filtration rate (eGFR). Additionally, they were asked to report adverse events, which might be brought by acupressure. The adverse events included faint or palpitation during acupressure, pain, nausea, and swelling of the ear.

### 2.6. Statistical Analysis

No previous trial was performed to study the effectiveness of acupressure for this condition; we assumed that acupressure would be of large effect size compared to no acupressure treatment. Therefore, we anticipated an effect size of 0.8 in this prospective trial. With significance level of 0.05 and study power of 0.8, a total sample size of 52 participants was needed. Considering a dropout rate of 20%, we included 62 participants.

Baseline values were described in Table 1. Continuous variables were presented as mean and standard deviation (SD), while categorical variables were shown as number of counts or percentages. For the primary outcome, we compared the difference between the 2 arms using analysis of covariance (ANCOVA) model. The model was adjusted for gender, baseline KDQOL-SF, and SF-36. Moreover, we calculated the effect size using the primary outcome through Cohen's *d* method, to examine whether our study is statistically powered. Cohen's *d* is defined as the difference between two means divided by a standard deviation for the data [27]. For the KDQOL-SF and SF-36 measured at different time points, we used the linear mixed effect model to detect the changes over time, as well as whether there was a significant difference between the 2 arms. We also adjusted this model for the same baseline values. Missing values were imputed using the propensity methods using the R software (http://www.r-project.com, version 3.1.0) [28]. Proportion of adverse events was compared between the 2 arms, using chi-square test. A *P* < 0.05 was considered a significant difference.

## 3. Results

One hundred and ninety-two participants were screened prior to this study, and 130 of them were excluded for violation of the inclusion criteria, unwillingness to participate, and other reasons. The included 62 participants were randomly allocated to the 2 arms, 31 in each arm. One participant in the control arm insisted on other complementary therapies after randomization and baseline assessment. One participant was lost to followup in the acupressure arm, while there were three in the control arm. We included all the 62 participants into statistical analysis, conforming to the intention-to-treat principle.

### 3.1. KDQOL-SF

**Figure 3:**
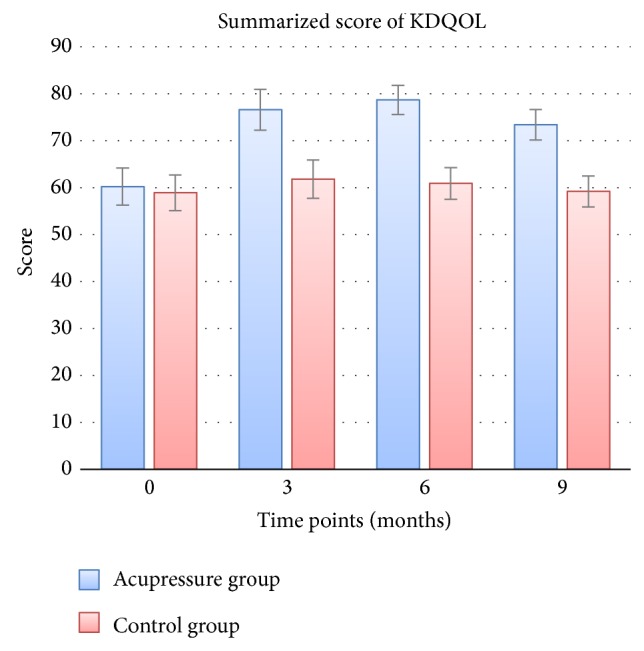
The primary outcome of this trial. This figure summarized the Kidney Disease and Quality of Life Short-Form (KDQOL-SF) at different assessment time points. Higher scores indicated better quality of life. The bars showed the mean scores of KDQOL-SF, while the error bars showed 95% confidential interval of the mean scores. The primary outcome (KDQOL-SF at 3 months after randomization) showed a significant difference between arms (*P* < 0.05). Similar results were found, when KDQOL-SF was assessed at 6 and 9 months after randomization.

**Figure 4:**
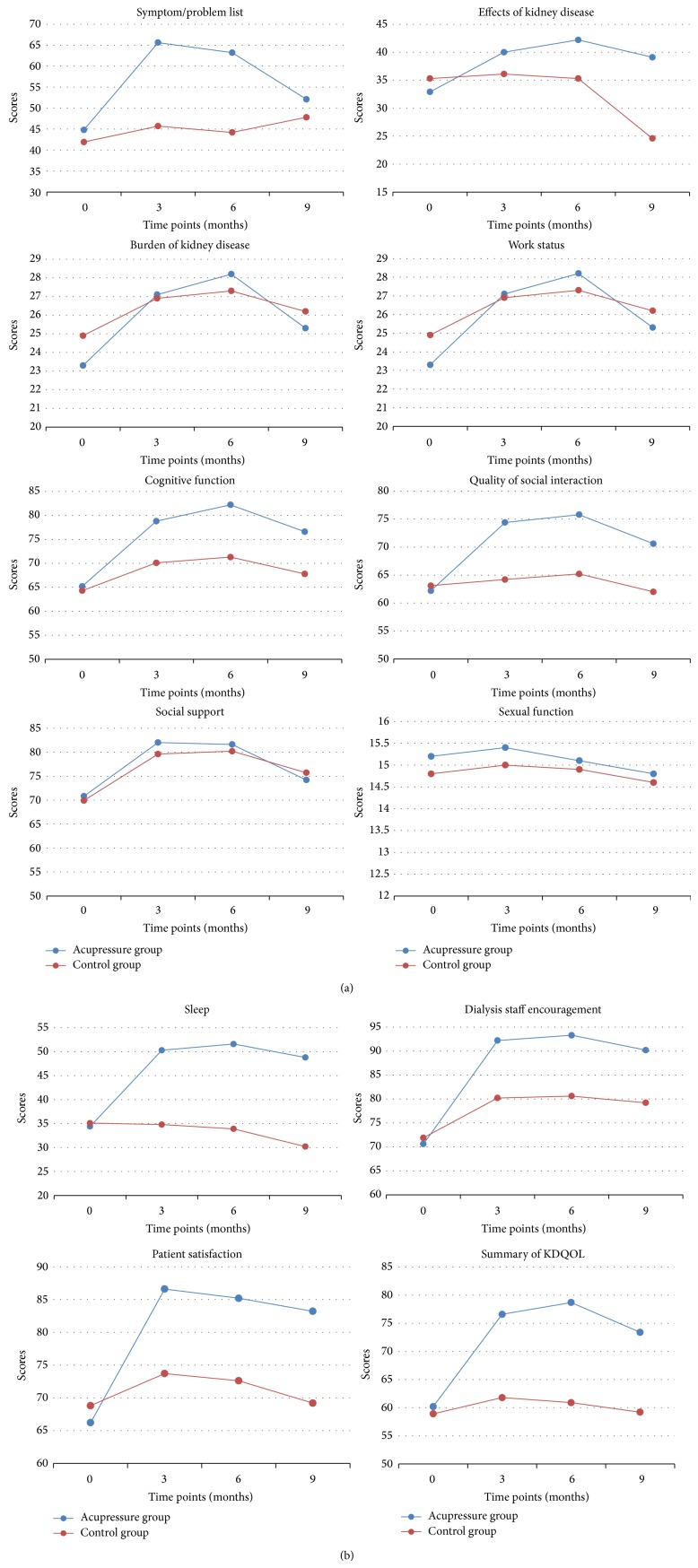
KDQOL-SF assessed at different time points. KDQOL-SF is short for Kidney Disease and Quality of Life Short-Form, which consisted of 11 items. We demonstrated the 11 items and a summarized score assessed at different time points.

KDQOL-SF was a specific assessment of quality of life in patients with chronic kidney diseases, and a higher score of KDQOL-SF indicated a better quality of life. The primary outcome was the summarized score of KDQOL-SF at 3 months after randomization. The results showed that patients in the acupressure arm reported significant higher KDQOL-SF scores compared to baseline (baseline versus 3 months after randomization, 60.2 versus 76.6, *P* < 0.001), but KDQOL-SF in the control arm did not improve significantly (baseline versus 3 months after randomization, 58.9 versus 61.8, *P* = 0.274). The primary outcome was 76.6 (95% CI, 72.2 to 81.0) in the acupressure arm, as compared to 61.8 (95% CI, 57.7 to 65.9) in the control arm (*P* < 0.001). The effect size of auricular acupressure towards no auricular acupressure was 1.48, calculated using the method of Cohen's *d*.  Figure 3 showed that, after 3 months of acupressure treatment, the summarized score of KDQOL-SF rose at 6 and 9 months after randomization. Moreover, similar results were found as the primary outcome when compared with the control arm.  Figure 4 showed the 11 items of KDQOL-SF assessment, 7 of which showed the same change as the primary outcome.

### 3.2. SF-36

**Figure 5:**
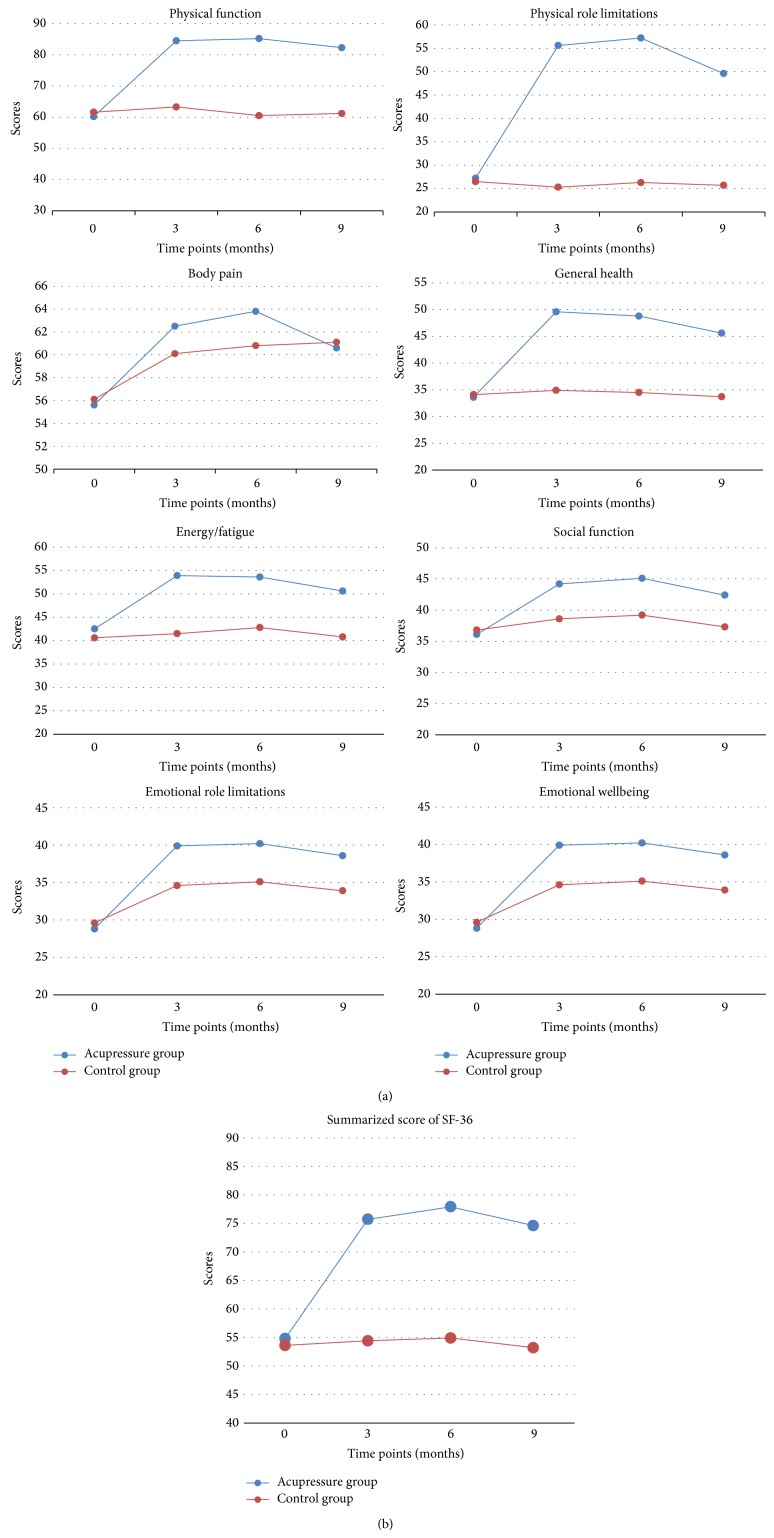
SF-36 assessed at different time points. SF-36 is short for 36-Item Short Form Health Survey, which consisted of 8 items. We showed the 8 items and a summarized score assessed at different time points.

SF-36 was an overall assessment for mental and physical conditions. A higher score of SF-36 also indicated a better outcome. The total score of SF-36 at 3 months after randomization was 75.7 in the acupressure arm (95% CI, 71.5 to 79.9), as compared to 54.4 in the control arm (95% CI, 51.3 to 57.6; *P* < 0.001). Similar results were found when SF-36 was assessed at 6 and 9 months after randomization. More details were shown in Figure 5.

### 3.3. HbA1c and eGFR

HbA1c reflected the blood sugar control in diabetic patients during the past 3 months. At 3 months after randomization, the HbA1c was 8.9% in the acupressure arm (95% CI, 7.8% to 10.0%), as compared to 8.3% in the control arm (95% CI, 7.0% to 9.6%; *P* = 0.536). The eGFR reflected the renal function; higher eGFR indicated a better outcome. After 3-month treatment, the eGFR was 15.2 in the acupressure arm (95% CI, 10.9 to 19.5), as compared to 14.9 in the control arm (95% CI, 11.0 to 18.8; *P* = 0.887).

### 3.4. Adverse Events

Two adverse events were reported by 2 participants in the acupressure arm, while no adverse event was reported in the control arm (*χ*-squared = 0.516, *P* = 0.472). One participant reported pain in the ear; the participant reported a score of 5 cm in visual analog scale (VAS) for the pain, which was a median intensity of pain. The pain disappeared after hot compress. The other participant reported nausea and palpitation during treatment; no vomiting was complained. The symptoms were relieved at about 2 hours after removing cowherb seed attachment. Therefore, we classified these adverse events as mild.

## 4. Discussion

This study showed that auricular acupressure was effective and safe for improving quality of life (QOL) in diabetic patients with chronic kidney disease (DCKD). To the best of our knowledge, this was the first study focusing on improving QOL of diabetic patients with chronic kidney disease. The result of this study revealed a new potential treatment option for this condition.

The effect size of auricular acupressure was nearly 1.5, which is large and promising for improving QOL in DCKD [29]. This effect size was calculated through the primary outcome (summarized score of KDQOL-SF at 3 months after randomization), using Cohen's *d* method. The effect size is rarely found and indicates a nonoverlap of 93% in the two arms, since Cohen defined the magnitudes of effect sizes as “small, *d* = 0.2,” “medium, *d* = 0.5,” and “large, *d* = 0.8.” Several reasons are considered to explain this effect size. First, we thought that it might be a placebo effect, which could be seen in trials using self-report outcomes [30, 31], and we used a self-reported scale (the KDQOL-SF) as the primary outcome for this study. Moreover, acupuncture related therapies induced larger placebo effect than other placebos, such as placebo drugs [32]. So the placebo effect might have played an important role in the large effect size of auricular acupressure in this study. Second, a good doctor-patient relationship might also contribute to the large effect size. In our study, the participants were educated by an acupuncturist on how to apply acupressure and were followed up to check if the manipulation of acupressure was correct. All these procedures might lead the patients to rate a better outcome than it actually was. Third, the large effect size might be caused by the fact that we only used quantitative measurements to assess QOL. We found that participants reported results of KDQOL-SF and SF-36 for relatively small variations in this study, which contributed to the large effect size owing to the small standard deviations. This could be caused by misunderstanding of the scales, which could be prevented by reconfirmation using qualitative measurements. However, we did not include a qualitative measurement for the concern of difficulty in interpreting the results. Qualitative methods might make an important contribution to randomized controlled trials for assessing complex health service interventions [33], such as acupuncture related therapies, so a mix of quantitative and qualitative measurements for future studies is warranted for addressing the above and the questions below. At last, this large effect size could also be a real effect of acupressure. However, as we aimed to testify the effectiveness of acupressure in this study, we did not include a sham control, which warrants a future study with an explanatory design.

There are 4 questions that remain unsolved after this study. First, we need a result of proportion of responders after finishing treatment, to get a global view of the effectiveness of auricular acupressure for the DCKD patients. Second, we need to address the frequency that would be the best for the patients to meet the acupuncturists. We still lack data to clarify whether the patients in our study were satisfied with meeting acupuncturists once every 2 weeks. Third, we need to address what are the appropriate treatment duration and the treatment protocol that will ensure the best adherence of the patients. Last, we need to find out how the self-managed auricular acupressure is perceived by the patients, which is closely related to the effectiveness of the acupressure intervention and the adherence to the acupressure protocol [34]. These questions were not well addressed in this study, so we still need a large sample size trial to confirm the effectiveness of auricular acupressure for DCKD patients and also to test the applicability in clinical practice.

In this study, we used the seeds of cowherb to stimulate the auricular points, instead of acupuncture needles, so the adverse events were rarely found. However, we found that a stimulating intensity of auricular acupressure should be standardized and well educated to the patients, since a participant complained of pain in the ear because of pressing hardly. A hard pressing may cause an unendurable pain that induces cardiovascular events, because the systems controlling cardiovascular function are closely coupled to the systems modulating the perception of pain [35].

We found an interesting result that auricular acupressure was not beneficial in controlling the level of HbA1c, which is contrary to the result of previous studies. Moreover, auricular acupressure was not helpful in increasing eGFR. These results might indicate that level of HbA1c and eGFR were not related to the QOL of patients with DCKD, which is consistent with the previous trials [36, 37]. However, this assumption might not be correct, because all the participants received intensive control of blood sugar and hemodialysis, and the parameters of HbA1c and eGFR were well treated.

There were several limitations in this study. First, the protocol of acupressure might not be the best and the most frequently used. The acupuncturist in this study developed the protocol, by her own clinical experience combined with the acupuncture textbooks. So we might have underestimated the effect size of acupressure to some extent. In the next trial with large sample size, we will consider a systematic review of acupuncture protocols before carrying out the trial. Additionally, we will consult several acupuncture experts in different provinces in China. Second, we did not control the placebo effect. Before this trial, no evidence was available as to whether auricular acupressure was effective or applicable to DCKD patients. So we aimed to answer the question of effectiveness first and then to calculate the effect size. Moreover, multiple comparisons in a small sample size trial would easily bring type I error. However, to determine the real effect (efficacy) of acupressure for DCKD patients, we should set a sham acupressure control, so the efficacy of auricular acupressure will be our next concern in this topic. Third, we did not assess the long term cardiovascular or death events in this study, so we could not answer the question of whether auricular acupressure is helpful in these events. DCKD patients had more cardiovascular or death events than non-DCKD patients, especially those who did not know about the harm of DCKD and took no treatments [38]. So reducing these events was also a major concern in DCKD patients, besides improving QOL. Fourth, the participants we included were all Chinese. So this result might not be generated to other populations. We hoped that we could include participants from different parts of the world to increase the generalizability of auricular acupressure.

## 5. Conclusion

Results of this study indicated that auricular acupressure significantly helped patients with chronic kidney diseases to improve their quality of life outcomes. Further quantitative and qualitative research is needed to confirm that self-managed acupressure is effective in patients living with a chronic disease.

## Figures and Tables

**Table 1:** Baseline characteristics of the included participants.

Parameters	Auricular arm (*n* = 31)	Control arm (*n* = 31)
Age (Year)	77.8 ± 8.5	75.7 ± 9.8
Men (%)	65.7	57.1
SBP (mmHg)	158.5 ± 15.9	159.8 ± 18.7
DBP (mmHg)	79.5 ± 11.5	75.5 ± 14.2
Weight (Kg)	62.5 ± 10.6	63.1 ± 6.7
Glycosylated hemoglobin (%)	8.7 ± 1.6	9.0 ± 1.3
Blood urea nitrogen (mmol/L)	27.8 ± 7.0	29.6 ± 7.3
Blood creatinine (umol/L)	665.3 ± 284.8	620.9 ± 265.6
Hemoglobin (g/dL)	9.9 ± 1.7	9.4 ± 1.6
Albumin (g/L)	2.8 ± 0.5	2.9 ± 0.5
eGFR (mL/min/1.73 m^2^)	10.2 ± 5.8	9.5 ± 6.2
Serum triglyceride (mmol/L)	2.0 ± 0.7	2.1 ± 0.5
Serum cholesterol (mmol/L)	5.2 ± 1.3	5.4 ± 1.6
LVDd (mm)	54.3 ± 8.1	52.5 ± 6.6
LVDs (mm)	36.2 ± 6.2	37.6 ± 5.7
LVEF (%)	51 ± 8	50 ± 10
NYHA		
III (*n*, %)	17 (68)	20 (80)
IV (*n*, %)	8 (32)	5 (20)
